# Emerging role of human polyomaviruses 6 and 7 in human cancers

**DOI:** 10.1186/s13027-021-00374-3

**Published:** 2021-05-17

**Authors:** Faisal Klufah, Ghalib Mobaraki, Dan Liu, Raed A. Alharbi, Anna Kordelia Kurz, Ernst Jan M. Speel, Véronique Winnepenninckx, Axel zur Hausen

**Affiliations:** 1 Department of Pathology, GROW-School for Oncology & Developmental Biology, Maastricht University Medical Centre+, Maastricht, the Netherlands; 2 Department of Laboratory Medicine, Faculty of Applied Medical Sciences, Albaha University, Albaha, Saudi Arabia; 3 Department of Medical Laboratories Technology, Faculty of Applied Medical Sciences, Jazan University, Jazan, Saudi Arabia; 4 Department of Hematology, The Affiliated Hospital of Southwest Medical University, Luzhou, China; 5 Department of Internal Medicine IV, RWTH Aachen University Hospital, Aachen, Germany

**Keywords:** HPyV6, HPyV7, Merkel cell polyomavirus, Oncogenic viruses, Cutaneous tumors, Viral persistence, Tumor virus

## Abstract

**Background:**

Currently 12 human polyomaviruses (HPyVs) have been identified, 6 of which have been associated with human diseases, including cancer. The discovery of the Merkel cell polyomavirus and its role in the etiopathogenesis in the majority of Merkel cell carcinomas has drawn significant attention, also to other novel HPyVs. In 2010, HPyV6 and HPyV7 were identified in healthy skin swabs. Ever since it has been speculated that they might contribute to the etiopathogenesis of skin and non-cutaneous human cancers.

**Main body:**

Here we comprehensively reviewed and summarized the current evidence potentially indicating an involvement of HPyV6 and HPyV7 in the etiopathogenesis of neoplastic human diseases. The seroprevalence of both HPyV6 and 7 is high in a normal population and increases with age. In skin cancer tissues, HPyV6- DNA was far more often prevalent than HPyV7 in contrast to cancers of other anatomic sites, in which HPyV7 DNA was more frequently detected.

**Conclusion:**

It is remarkable to find that the detection rate of HPyV6-DNA in tissues of skin malignancies is higher than HPyV7-DNA and may indicate a role of HPyV6 in the etiopathogenesis of the respected skin cancers. However, the sheer presence of viral DNA is not enough to prove a role in the etiopathogenesis of these cancers.

## Background

Polyomaviruses (PyV’s) comprise a family of non-enveloped viruses containing a small genome of approximately 5.0 kbp in size of circular double-stranded DNA and are capable of infecting mammals and birds [1–3]. PyV’s are strongly oncogenic in heterologous animal models, suggesting that next to human also non-human polyomaviruses could possibly play a role in human cancers [4–7]. To date, fifteen novel PyV’s have been isolated from different human specimens [8]. In 2019, the International Committee on Taxonomy of Viruses (ICTV) reported only twelve human polyomaviruses (HPyVs) after having HPyV12 excluded, which was shown to be infecting shrews [9, 10]. In addition, the Lyon IARC polyomavirus which initially was isolated from human skin swab in 2017 is also found in cats feces [11]. Recently in 2019, Ondov et al. isolated the Quebec PyV from a stool sample of one patient through the MinHash algorithm [12]. Yet it still has not been listed as a human polyomavirus by the ICTV [9]. Six of the identified HPyVs have been associated with human diseases, including cancer [3, 8, 13, 14].

In 2008, Merkel cell polyomavirus (MCPyV) has been linked to the etiopathogenesis of the majority (i.e. 80%) of the highly aggressive neuroendocrine Merkel cell carcinoma (MCC) [15–17]. Already in 2012 MCPyV has been categorized as a group 2A carcinogen by the International Agency for Research on Cancer (IARC) [18]. MCPyV has also been detected in non-neoplastic B cells (e.g. reactive hyperplasia and normal lymph node) and neoplastic B cells (e.g. chronic lymphocytic leukemia cells,) suggesting a role for MCPyV in B-cell neoplasias [15, 19–22].

In 2010, HPyV6 and HPyV7 were identified from healthy skin swabs using the rolling circle amplification technique [23]. The HPyV7 genome shares approximately 68% sequence identity with HPyV6 [23]. As these novel HPyVs were discovered in the skin, their presence in the skin and non-cutaneous human cancers, similar to MCPyV has been studied intensively [24–31]. Additionally, HPyV6 and 7 proteins have been suggested to play a key role in the binding and deactivation of p53 [32]. The oncogenic role of HPyVs in general has been reviewed extensively [3, 7, 13, 33–36]. By reviewing sero-epidemiological and tumor-pathological evidence, we here focus on the possible oncogenic role of HPyV6 and 7 in human cancers, and in particular their direct or indirect contribution to tumorigenesis.

### Seroprevalence of HPyV6 and HPyV7 in healthy individuals

Several studies have assessed HPyV6 and HPyV7 serum antibody levels across a broad range of age groups of various populations [23, 37–41]. In 2010, 95 sera from blood donors were tested for the seroprevalence of HPyVs and revealed higher seropositivity for HPyV6 (69%) than HPyV7 (35%) using direct enzyme-linked immunosorbent assays (ELISA) directed against virus-like particles (VLP) based on viral protein 1 (VP1) capsid [23]. Likewise, by using ELISA against VP1-VLP, another study estimated the seroprevalence of age groups ranging from 20 years and older and concluded that the prevalence of HPyV6 increased with age and was higher than that of HPyV7 (80–92% vs 60–80%, respectively) [37]. The HPyVs seroprevalence of 1050 Dutch blood donors aged from 18 to 69 was determined and showed that the prevalence of both viruses increased with age, with 84 and 72% seropositivity for HPyV6 and HPyV7 VP1 antibodies, respectively [38]. Nicol et al. assessed the seroprevalence of both viruses in 828 subjects across different age groups starting from age one using VLP-based direct ELISA. Their study found that HPyV6 (76%) was more prevalent than HPyV7 (53%) across all age groups [39]. Noteworthy, by performing Luminex xMAP technology to detect VP1antibodies for both viruses were detected in the early infancy group up to an age of 6 months with high frequency (80.1% for HPyV6 and 58.1% for HPyV7, respectively). On the other hand, a decline in the prevalence of both viruses was found for children age 6 months to 14 years old group [40].

**Fig. 1:**
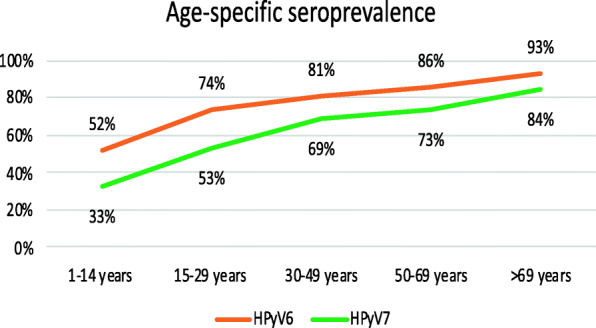
Summarizing age-specific seroprevalence overall positivity of HPyV6 and HPyV7 using ELISA or Luminex platform-based methods to analyze antibodies against virus-like particles and/or glutathione-S-transferase consisting HPyV6 and 7 viral protein

Altogether, both HPyV6 and 7 were found to be ubiquitous and to infect all age groups, and HPyV6 seroprevalence is slightly higher than HPyV7. Seroprevalence significantly increases with age, and a substantial proportion of individuals 50 years of age and older has tested positive for HPyV6 and HPyV7. Approximately 52–93% of humans are seropositive for HPyV6, whereas 33–84% are seropositive for HPyV7 according to the seroprevalence studies Table 1 [23, 37–40, 42, 43]. Summarizing the overall age-specific seropositivity of HPyV6 and HPyV7 using ELISA or Luminex platform-based method was shown in (Fig. 1).

**Table 1:** Seroprevalence of HPyV6 and HPyV7 antibodies in serum using ELISA or Luminex platform

Source of the specimen	Age (y)	Samples n	HPyV6 Positive n (%)		HPyV7 Positive n (%)		Assay	Reference
			VLP	GST	VLP	GST		
18–65 (y) Blood donors. < 18 or > 65 (y) From discarded lab samples	1–4	48	18 (37.5)		5 (10.4)		ELISA	[39]
	5–9	69	40 (57.9)		23 (33.3)			
	10–14	92	65 (70.6)		41 (44.6)			
	15–19	89	55 (61.8)		32 (36)			
	20–29	49	43 (87.8)		22 (44.9)			
	30–39	73	49 (67.1)		31 (42.5)			
	40–49	105	89 (84.8)		62 (59.1)			
	50–59	95	78 (82.1)		64 (67.4)			
	60–69	100	88 (88)		68 (68)			
	70–79	52	46 (88.5)		42 (80.8)			
	≥ 80	56	55 (98.2)		48 (85.7)			
Blood donors	20–29	96	82 (85.4)	77 (80.2)	58 (60.4)	68 (70.8)	ELISA	[37]
	30–39	129	114 (88.4)	111 (86)	92 (71.3)	106 (82.2)		
	40–49	52	48 (92.3)	48 (92.3)	32 (61.5)	40 (76.9)		
	50–59	24	22 (91.7)	21 (87.5)	16 (66.7)	19 (79.2)		
	> 59	5	4 (80)	4 (80)	3 (60)	4 (80)		
Serum obtained for routine lab tests	0–0.5	31		25 (80.6)		18 (58.1)	Luminex platform	[40]
	0.6–1.9	63		14 (22.2)		12 (19)		
	2–3	62		21 (33.9)		12 (19.4)		
	4–5	58		26 (44.8)		19 (32.8)		
	6–7	58		29 (50)		21 (36.2)		
	8–9	70		44 (62.9)		27 (38.6)		
	10–14	55		31 (56.4)		21 (38.2)		
	15–19	59		38 (64.4)		25 (42.4)		
	20–29	59		39 (66.1)		30 (50.8)		
	30–39	64		41 (64.1)		35 (54.7)		
	40–49	54		34 (63)		33 (61.1)		
	50–59	58		46 (79.3)		36 (62.1)		
	60–69	54		50 (92.6)		45 (83.3)		
	> 70	54		50 (92.6)		46 (85.2)		
Blood donors	18–29	208		156 (75.7)		117 (56.8)	Luminex platform	[38]
	30–39	209		175 (84.5)		154 (74.4)		
	40–49	208		173 (83.6)		155 (74.9)		
	50–59	215		184 (85.6)		154 (71.6)		
	60–69	210		187 (89.5)		169 (80.9)		
Blood donors		95	66 (69)		33 (35)		ELISA	[23]
SCCs transplanted cases		110		65 (59)		57 (51.8)	ELISA and Luminex platform	[42]
Non-SCCs transplanted cases		207		137 (66.2)		85 (41)		
Lung cancer		511	393 (76.9)		333 (65.2)		Luminex platform	[43]
Controls		508	400 (78.7)		334 (65.8)			

*ELISA* enzyme-linked immunosorbent assays, *GST* glutathione-S-transferase, *SCC* squamous cell carcinoma, *VLP* virus-like particles, (*y*) years old

In general, HPyV6 and 7 infections seem to be clinically rather asymptomatic, irrespective of the age at which the infection occurs. Increasing seroprevalence of these viruses is clearly age-related and reaches its peak in individuals beyond 40 years of age, possibly indicating a relationship of HPyV6 and 7 infections with immunosenescence. Regarding the methodology, the previous studies reported the seroprevalence of HPyV6 and HPyV7 in the sera obtained from blood donors or routine lab tests in various studies. Most of the studies used ELISA or Luminex platform antibody-binding assay methods to detect the antigen containing the structural viral capsid protein of the targeted viruses fused with VLP or glutathione S-transferase (GST) proteins Table 1. Moreover, one study assessed the agreement and sensitivity of both VLP and GST-based ELISA methods in detecting seropositivity [37]. The results showed only minor discrepancies in the seroprevalence comparing these two detection methods [37]. Kamminga et al. reported high seropositivity of HPyV6 and 7 by using a Luminex xMAP assay directed against the VP1 capsid of HPyVs, similar to other ELISA-based studies [37, 39–41].

### Seroprevalence of HPyV6 and HPyV7 in cancer patients

Only a very limited number of studies have reported seroprevalence data of HPyV6 and 7 in cancer patients. Madeleine et al. recently reported that the seroprevalence of HPyV6 in immunosuppressed skin cancer patients (59.1%) was lower compared to healthy controls (66.2%), whereas the seroprevalence of HPyV7 in the same patient cohort was slightly elevated (52%) compared to the control group (41%) [42]. It is important to note that the ELISA GST detection method used by Madeleine et al. yielded a much lower seroprevalence compared to other studies using the same technique. Similarly, serum from non-smoking-related lung cancer patients showed positivity for HPyV6 (76.9%) and HPyV7 (65.2%), which was comparable to the seroprevalence of these viruses in normal population age groups older than 50 years [43]. HPyV6 and 7 seropositivity data and results, including methodology, are summarized in Table 1.

In the past, studies on the seroprevalence of other human tumor viruses, e.g. Epstein-Barr virus or human papillomaviruses (HPV), have helped to indirectly indicate whether or not these viruses are involved in the etiopathogenesis of human cancers [44–48]. Moreover, the infection by small DNA tumor viruses at an early age, such as high-risk HPV, has been identified as a potential cause of human cancer decades after infection [47–49]. Also, HPyV6 and 7 antibodies are already detectable in the sera of healthy individuals at an early age Table 1 indicating that a substantial number of individuals – if not the majority - acquire both viruses already during their early lifetime, which might be a prerequisite for the onset of a possibly associated disease or cancer later in life.

### Prevalence of HPyV6 and HPyV7 DNA in non-neoplastic diseases

A limited number of studies have screened for the prevalence of HPyV6- and HPyV7-DNA in non-neoplastic diseases, with very low prevalence across a variety of specimens Table 2. Both HPyVs were reported in Kimura’s disease - a chronic inflammatory disorder associated with lymphadenopathy - with 4/5 and 1/5 samples found to be positive for HPyV6- and HPyV7-DNA, respectively [50]. Only HPyV6-DNA was detected in lichen simplex chronicus (1/13 samples), Kikuchi disease, i.e. histiocytic necrotizing lymphadenitis (3/8 samples), dermatopathic lymph-adenitis (1/28 samples), and angiolymphoid hyperplasia with eosinophilia (ALHE) (4/5 samples) [24, 54, 55]. In addition, only one study showed the presence of HPyV6-DNA in lymphocytes of lymph nodes tissue from ALHE case by fluorescence in situ hybridization (FISH) [54].

**Table 2:** Prevalence of HPyV6 and HPyV7 in human non-neoplastic diseases

Non-neoplastic disease	Specimen	Samples n	Method	HPyV6 Positive n (%)	HPyV7 Positive n (%)	Reference
Cutaneous diseases						
Dyskeratosis and parakeratosis	FFPE	3	PCR/qPCR/IHC/ Seq.	2 (66.6)	1 (33.3)	[50]
Lichen simplex chronicus	FF	13	rt-PCR	1 (7.7)	ND	[24]
Pruritic in Cardiac transplant recipient	FFPE	1	IHC/PCR	NA	1 (100)	[51]
Pruritic Rash (lung transplant)	FFPE	2	IHC/PCR/RCA PCR/rt-PCR	NA	2 (100)	[52]
Viral warts	FFPE	12	qPCR	3 (25)	8 (66.7)	[53]
Angiolymphoid hyperplasia with eosinophilia or Kimura disease^a^	FF	1	Shotgun metagenomic/FISH	1 (100)	NA	[54]
Angiolymphoid hyperplasia with eosinophilia	FFPE	5	qPCR/IHC	4 (80)	0	[55]
Kimura disease	FFPE	5	qPCR/IHC	4 (80)	1 (20)	[55]
Kikuchi disease	FFPE	38	qPCR	3 (8)	0	[55]
Dermatopathic lymphadenitis	FFPE	28	qPCR	1 (4)	0	[55]
Non-cutaneous diseases						
Thymic hyperplasia	FFPE	20	PCR/IHC/FISH	NA	8 (40)	[31]
Chronic tonsillitis & tonsillar hypertrophy	FF	78	Luminex-assay	6 (7.7)	ND	[56]
Chronic tonsillitis & tonsillar hyperplasia	FF	40	qPCR	1 (2.5)	ND	[57]
Tonsillectomy (due to chronic tonsillitis, peritonsillar abscess, or sleep apnea syndrome)	FF and FFPE	108	qPCR	5 (4.6)	1 (0.9)	[58]
Patients with respiratory symptoms	NPA	1232	rt-PCR	2 (0.16)	3 (0.24)	[59]
Respiratory tract infections	NPA	887	rt-PCR	15 (1.7)	ND	[60]
HIV patients with leukoencephalopathies.	CSF	14	NGS/PCR	1 (7.1)	NA	[61]
Meningoencephalitis	CSF	135	qPCR	1 (0.74)	0	[62]
Immunocompromised patients scheduled to receive transplants	NPS, urine, fecal, plasma	32	rt-PCR	3 (9.4)	1 (3.1)	[63]
Acute gallstone cholangitis	Bile fluids	91	rt-PCR	5 (5.5)	NA	[64]
Patients with gastrointestinal illness	Fecal	185	rt-PCR	1 (0.5)	1 (0.5)	[59]

^a^ALHE case with Kimura disease clinical syndromes

*FF* fresh frozen tissue, *FFPE* formalin-fixed paraffin-embedded, *FISH* fluorescence in situ hybridization, *IHC* immunohistochemistry, *NA* not applicable, *ND* not detected, *NGS* next-generation sequencing, *NPA* nasopharyngeal aspirates, *NPS* nasopharyngeal swab, *PCR* polymerase chain reaction, *qPCR* quantitative polymerase chain reaction, *rt-PCR* real-time polymerase chain reaction, *Seq* Sanger sequencing

Studies conducted on patients with cutaneous diseases have reported the presence of both HPyV6- and 7-DNA in tissue specimens. Three dyskeratotic dermatosis cases tissue revealed 2 positives for HPyV6 and 1 positive for HPyV7, and in previously 12 HPV-induced warts tested showed that 3 positives for HPyV6 and 8 for HPyV7 [50, 53]. In addition, HPyV7-DNA was found in all three biopsies of pruritic patients, thus the diagnostic term HPyV 7-associated pruritic rash has been introduced in transplantation medicine [51, 52]. The higher prevalence of both viruses in skin disease samples could be attributed to their high occurrence in the skin of healthy individuals. However, the viral load and prevalence of both viruses seem to increase in the skin of elderly people, in particular immunosuppressed individuals, which may lead to associated skin disorders [65].

The prevalence of HPyV6- and 7-DNA in non-neoplastic non-cutaneous diseases appears to be much lower compared to non-neoplastic skin diseases. Our group reported the presence of HPyV7-DNA in (3/8; 40%) of non-malignant thymic hyperplasia [31]. Other studies detected HPyV6-DNA in (12/226; 5.3%) of tonsillitis tissue specimens Table 2 [56–58]. The detection rate of both viruses was found to be slightly higher in tissue specimens in contrast to body fluids specimens [24, 31, 50–54, 56–61, 63, 64]. For instance, in cerebrospinal fluid, HPyV6-DNA was detected in (1/14; 7.1%) samples from HIV patients with leukoencephalopathies and (1/135; 0.7%) samples from patients with meningoencephalitis [61, 62]. In addition, (5/91; 5.5%) bile fluid samples from acute gallstone cholangitis patients tested positive for HPyV6 [64]. In comparison, there was a low detection rate (< 2%) of both viruses in nasopharyngeal aspirates specimens collected from respiratory symptomatic patients [59, 60]. Similarly, the presence of HPyV6 and 7 was uncommon (< 1%) in the feces obtained from patients with gastrointestinal illness for routine lab investigation [59]. Overall, the prevalence of HPyV6 and HPyV7 in formalin-fixed-paraffin-embedded (FFPE) and fresh frozen biopsies were elevated compared to liquid biopsy specimens, in which the prevalence of HPyV6 and HPyV7 was 1.1 and 0.2%, respectively Table 2.

### HPyV6 and HPyV7 DNA prevalence in primary cutaneous malignancies

According to the World Health Organization, the incidence of skin tumors has increased over the past decades, approximately 8500 new cases of skin cancers are reported daily in the United States [66, 67]. Up to date, the presence of the DNA of five HPyVs has been reported in the human skin: MCPyV, HPyV6, HPyV7, HPyV9, and trichodysplasia spinulosa–associated polyomavirus [40]. Of these, only MCPyV has been identified as a novel human tumor virus closely linked to the etiopathogenesis of the majority of MCCs. Since HPyV6 and 7 were isolated from the skin their possible contribution to the etiopathogenesis of skin cancers has been studied intensively [24–30, 68]. Numerous studies, summarized in Table 3, have assessed the prevalence of HPyV6- and 7-DNA in primary cutaneous malignancies, including epithelial, neuroendocrine, and lymphoid skin cancers.

**Table 3:** Prevalence of HPyV6 and HPyV7 in primary cutaneous malignancies

Cancer	Specimen types	Samples n	Method	HPyV6 Positive n (%)	HPyV7 Positive n (%)	Reference
MM	FFPE	47	rt-PCR/Seq.	2 (4.3)	2 (4.3)	[25]
AK	FFPE	31	qPCR	1 (3)	ND	[27]
AK	FFPE	92	Seq.	4 (4.3)	NA	[69]
AK	FFPE	2	qPCR	ND	1 (50)	[53]
VK	FFPE	4	qPCR	3 (75)	3 (75)	[53]
BCC	FFPE	18	qPCR	10 (5.5)	ND	[29]
BCC	FFPE	41	qPCR	3 (7)	ND	[27]
BCC	FFPE	50	rt-PCR/Seq.	1 (2)	2 (4)	[25]
BCC	FFPE	109	PCR/Seq./FISH	23 (21.1)	NA	[68]
SCC	FFPE	8	qPCR	1 (12)	ND	[27]
SCC	FFPE	21	qPCR	8 (38)	ND	[29]
SCC	FFPE	52	qPCR	2 (4)	ND	[27]
SCC	FFPE	86	PCR/Seq./FISH	8 (9.3)	NA	[68]
SCC	FFPE	63	rt-PCR/Seq.	2 (3.2)	1 (1.6)	[25]
SCC IN CLL cases who had BMT	FFPE	3	Bead-based multiplex PCR	1 (33.3)	ND	[28]
SCC	FFPE	11	qPCR	4 (36.4)	6 (54.5)	[53]
SCC	FFPE	17 (6 patients)	rt-PCR/IHC	17 (100)	17 (100)	[26]
KA	FFPE	42	qPCR	2 (5)	ND	[27]
KA	FFPE	59	PCR/Seq/FISH	25 (42.3)	NA	[68]
TB	FFPE	45	PCR/Seq	10 (22.2)	NA	[68]
MCC	FFPE	20	qPCR	2 (10)	ND	[29]
MCC	FFPE	28	rt-PCR	1 (3.5)	1 (3.5)	[30]
CTCLs	FFPE	116	rt-PCR	6 (5.20)	1 (0.90)	[70]
CTCLs	FF	71	rt-PCR	13 (18.3)	ND	[24]
CTCLs	FF and FFPE	35	rt-PCR	7 (20)	3 (8.6)	[71]
Total		1072		157/1072 (14.6)	37/681 (5.4)	

*AK* actinic keratosis, *BCC* basal cell carcinoma, *BMT* bone marrow transplantation, *CLL* chronic lymphocytic leukemia, *CTCLs* cutaneous T-cell lymphomas, *FFPE* formalin-fixed paraffin-embedded, *FISH* fluorescence in situ hybridization, *IHC* immunohistochemistry, *FF* fresh frozen tissue, *KA* keratoacanthoma, *MCC* Merkel cell carcinoma, *MM* malignant melanoma, *NA* not applicable, *ND* not detected, *PCR* polymerase chain reaction, *qPCR* quantitative polymerase chain reaction, *rt-PCR* real-time polymerase chain reaction, *SCC* squamous cell carcinoma, *Seq* Sanger sequencing, *TB* trichoblastoma, *VK* verrucous keratosis

One study reported the presence of HPyV6- and HPyV7-DNA (2/47; 4.3%) in malignant melanoma (MM) specimens using rt-PCR [25]. In non-melanoma skin cancer tissues, both HPyV6- and 7-DNA have also been identified. Four studies have shown the presence of HPyV6-DNA in the most common human skin cancer, i.e. basal cell carcinoma (BCC) Table 3. The detection rate was between 2 to 21%, and one study confirmed the detection of HPyV6 in BCC by FISH [25, 27, 29, 68]. HPyV7-DNA was identified in 4% of BCC cases by rt-PCR in a single study [25]. Studies on squamous cell carcinoma (SCC), which is the second most common human cutaneous malignancy, revealed a prevalence between 3 to 38% for HPyV6 [25, 27–29, 68]. Other studies tested keratoacanthoma and trichoblastoma for HPyV6-DNA, although no studies have tested trichoblastoma for HPyV7 [27, 68]. HPyV6- and 7-DNA were detected in < 2% of tissue biopsies from MCC and < 4% of actinic keratosis tissues [27, 29, 30, 53, 69]. To date, only one study has analyzed verrucous keratosis tissues, and (3/4; 75%) specimens were positive for both viruses [53]. Another study by Schrama et al. detected HPyV6 in all tested 18 epithelial proliferation specimens (SCC and/or keratoacanthoma obtained from MM patients treated with the BRAF inhibitor vemurafenib) and HPyV7 in 17/18 specimens using rt-PCR combined with immunohistochemistry (IHC) using 6V32 and 6V12 antibodies targeting HPyV6 VP1 and have cross-reactivity with HPyV7 [26]. It is of interest to mention that only very few studies report the usage of IHC to screen for HPyV6 and 7 protein expression, due to the lack of commercially available HPyV6- or 7-specific antibodies. In this respect, one may also consider the use of the commonly PAb416 antibody used in routine laboratories originally directed against the LTAg of SV40. PAb416 antibody which detects conserved epitope region of diverse HPyVs including HPyV6 and 7 but not detecting MCPyV which there is a commercial antibody (CM2B4) available to detect its LTAg [72, 73].

Additionally, primary cutaneous lymphomas have been tested for the presence of HPyV6- and 7-DNA: cutaneous T-cell lymphoma (CTCL) patients, and all three studies showed that CTCLs were positive (5.20 to 20%) for HPyV6- and (0.90 to 8.6%) for HPyV7-DNA, while no cutaneous B-cell lymphoma was positive for either virus [24, 70, 71]. Overall, results showed that the occurrence of HPyV6-DNA in CTCL was more frequent than HPyV7-DNA Table 3.

**Fig. 2:**
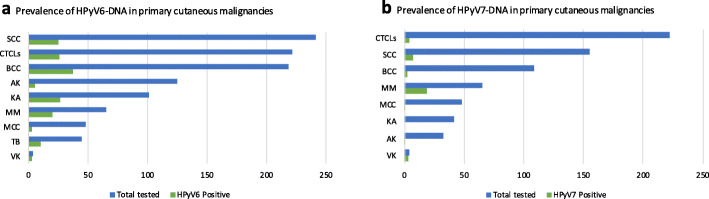
Representing a summary of the prevalence of HPyV6-DNA (**a**) and HPyV7-DNA (**b**) in tissues of cutaneous malignancies. AK, actinic keratosis; BCC, basal cell carcinoma; CTCLs, cutaneous T-cell lymphomas; KA, keratoacanthoma; MCC, Merkel cell carcinoma; MM, malignant melanoma; SCC, squamous cell carcinoma; TB, trichoblastoma; VK, verrucous keratosis

It is remarkable that HPyV6-DNA is far more often detected than HPyV7-DNA in human neoplastic skin diseases (Fig. 2), which may indicate a role for HPyV6 in the etiopathogenesis of these cancers. However, the reported copy loads of HPyV6- and 7-DNA in diverse skin cancers do not clearly support this hypothesis. Studies reported a generally low viral load of HPyV6- and 7-DNA-positive skin cancers which does not reflect the situation of high viral load of MCPyV in MCC [25, 27, 29, 53, 74]. Nevertheless, viral load copies of HPyV6 per cell range (0.00014–0.14) and MCPyV (0.0016–0.36) were almost in the same range in non-MCC tumors such as SCC, BCC, and MM, while HPyV7 copy numbers (0.000079–0.0094) were much lower. Moreover, specimens from healthy skin specimens showed the same range of HPyV6-DNA and MCPyV-DNA viral load as non-MCC samples [25, 27, 29, 53]. Notably, HPyV7-DNA was more often detectable than HPyV6-DNA in non-cutaneous cancers, while HPyV6-DNA was identified more often in skin malignancies Tables 2 and 3.

### HPyV6 and HPyV7 in non-cutaneous malignancies

The prevalence of HPyV6 and 7 have also been studied in a variety of non-cutaneous malignancies summarized in Table 4. In tissue specimens, HPyV7- but not HPyV6-DNA was frequently (62%) found in thymic epithelial tumors [31]. In contrast, HPyV6-DNA was more prevalent (5.4%) in tonsillar SCC [57, 58].

**Table 4:** Prevalence of HPyV6 and HPyV7 in non-cutaneous other human malignancies

Tumor	Specimen	Samples n	Method	HPyV6 Positive n (%)	HPyV7 Positive n (%)	Reference
BC	FF	54	rt-PCR	1 (2)	1 (2)	[75]
LSCC	FF	7	Luminex platform (Multiplex PCR)	1 (14.3)	ND	[76]
TETs	FFPE	37	FISH/PCR/Seq/IHC	ND	23 (62.2)	[31]
TSCC	FF	38	qPCR	2 (5.3)	1 (2.6)	[57]
TC	FF and FFPE	112	qPCR	6 (5.4)	2 (1.8)	[58]
CCA	FFPE	42	PCR/FISH/RISH/IHC	4 (9.5)	19 (45.2)	[77]

*BC* breast cancer, *CCA* cholangiocarcinoma, *FFPE* formalin-fixed paraffin-embedded, *FF* Freshly frozen tissue, *FISH* fluorescence in situ hybridization, *LSCC* laryngeal squamous cell carcinoma, *ND* not detected, *PCR* polymerase chain reaction, *qPCR* quantitative polymerase chain reaction, *rt-PCR* real-time polymerase chain reaction, *RISH* RNA fluorescence in situ hybridization, *Seq* Sanger sequencing, *TC* tonsillar carcinoma, *TETs* thymic epithelial tumors, *TSCC* tonsillar squamous cell carcinoma

In body fluids specimens, Chan et al. investigated the presence of HPyVs in the bile fluid of patients with a variety of hepatobiliary malignancies [64]. HPyV6 but not HPyV7 was detected in 27.6, 10.7, 16.7, and 20% of bile specimens from cholangiocarcinoma (CCA), hepatocellular carcinoma, pancreatic carcinoma, and gallbladder carcinoma patients, respectively [64]. We recently identified the presence of HPyV6- and 7-DNA in cholangiocarcinoma cases at the single-cell level by diverse molecular biology techniques for the detection of viral DNA, mRNA, and protein expression [77]. We found that HPyV7 was more prevalent than HPyV6 and indeed the presence of these viruses was not restricted to CCA tissues but also found in adjacent microscopically non-neoplastic hepatocytes [77].

Overall, among patients with different non-cutaneous cancers, HPyV7-DNA was more frequently detected than HPyV6-DNA in tissues. Yet, it remains unclear if and how HPyV7 might contribute to the tumorigenesis of these thymic epithelial tumors or cholangiocarcinomas, or that it rather is present in the context of viral latency.

### Latency and tropism

The frequent prevalence of HPyV6- and 7-DNA in non-neoplastic tissues along with their high seropositivity indicates that these viruses remain in long-term latency in humans. Therefore, it is important to unmask the host cells facilitating this long-term latency in healthy individuals to understand the host cell-type specific encoded proteins needed for viral entrance, replication, and transcription.

So far, the underlying mechanisms of tissue tropism or the possible latency of HPyV6 and 7 are still unknown. Other HPyVs have been reported to remain in latency within the epithelium of the proximal tubule of the kidney (BKPyV) while JCPyV is found in the brain [3]. In other studies, BKPyV and JCPyV were suggested to be found latent in lymphoid tissues and lymphocytes [78]. Since the seroprevalence of HPyV6 and 7 is high in the general population, it is difficult to ultimately determine the latency compartment of these two HPyVs based on the currently available literature. However, keratinocytes have been identified to be the potential primary target of HPyV7 infection and replication [50]. In addition, since HPyV 6 and 7 have been detected in BCC, further studies are needed to investigate whether they act similar to HPV by infecting the basal epithelial cells through lesions in the epidermis and undergo episomal replication in the parabasal layers [25, 27, 29, 68, 79].

### Direct versus indirect HPyVs-related carcinogenesis

The potential contribution of oncogenic viruses is either direct and/or indirect in the carcinogenesis of virus-associated tumors [80, 81]. MCPyV is the only HPyV found to be clonally integrated into the genome of human cancer, namely MCC. Next to its clonal integration, the MCPyV genome carries tumor-specific mutations within the large tumor antigen (LTAg) introducing stop codons and thus abrogating the helicase expression and rendering MCPyV replication-deficient [15, 17]. In direct carcinogenesis, one would expect a high expression of viral proteins in every tumor cell, which indeed is seen for the MCPyV-LTAg protein in MCPyV positive MCC [15, 17, 82]. In particular, the binding of truncated LTAg to the retinoblastoma protein-binding (RB) maintains the replication of MCPyV-positive MCC cells [3, 26, 83]. During the preparation of this review it occurred, that only a few studies report the protein expression of HPyV6 and 7 in neoplastic cells, possibly indicating such a direct tumorigenic role as shown for MCPyV and MCC. However, HPyV6- and 7-LTAg expression are not found in all tumor cells in the above mentioned possibly HPyV6 and 7-associated human cancers. Thus, it seems unlikely that both HPyVs play an important direct contribution to human tumorigenesis [26, 31, 77].

Human tumor viruses also can induce neoplasms by indirect mechanisms via the interaction between viral antigens, environmental factors, and other modifications to the host genome [80]. Inflammatory reactions due to the immune response to viral infection exposure can become eradicated and induce acute inflammation which may also result in chronic inflammation [84, 85]. Reactive oxygen radicals can be produced years later following chronic inflammation and trigger modifications in the host nucleic acids and then inducing transformation as it is known for hepatitis B and C viruses [84, 86]. For instance, the hepatitis C virus is not detected in the cancer cells that may develop after many years of infection but are found in the adjacent lesions to the hepatocellular carcinoma [87–90]. The infection by the virus alone is not sufficient to develop a tumor, and persistent viral infection precedes possibly many years before a cancer develops by an indirect mechanism such as chronic inflammation. The recent finding of HPyV6 and 7 in hepato- and cholangiocellular tissues possibly indicates such a physiopathologic mechanism of chronic inflammation-related indirect carcinogenesis also for HPyV6 and 7 [77]. However, more studies are needed to further elucidate and establish chronic inflammation-related indirect carcinogenesis for HPyV6 and 7.

Besides the direct and indirect HPyVs-related carcinogenesis, the “hit and run” hypothesis has been proposed to explain a possible role of viral agents to mediate cellular transformation [91]. The “hit and run” concept was proposed by Skinner in 1976 as a model where the virus is required to initiate the tumorigenesis process by leading to mutations, which maintain the tumorigenesis resulting in tumor with loss of the viral genes in the later stages [91]. Few studies have suggested the “hit and run” mechanism to mediate transformation by BKPyV, JCPyV, and MCPyV [92–96]. However, this hypothesis remains speculative until further evidence for “hit and run” for - HPyV associated diseases is established.

In addition, HPyVs may contribute to human tumorigenesis together with other oncogenic viruses assuming, that two or maybe more different viruses potentially infect the same cells and interact with each other in the etiopathogenesis of human cancers [97–100]. Primary evidence for interplay and helper functions of viruses had been established since long, especially for adenoviruses and herpesviruses [101, 102]. Of interest, Heilbronn et al. reported the finding that cytomegalovirus (CMV) infection can trigger JCPyV-DNA replication in cultured human fibroblasts [103]. Indirect potential evidence for possible interplay and helper functions is currently restricted to observational findings: BKPyV has been found to be associated with the induction of cervical intraepithelial neoplasias, particularly in immunosuppressed patients [104, 105]. Although these findings might point to an interaction of certain high-risk HPV types with BKPyV, they are by far no causal proof that BKPyV contributes to the HPV-related etiopathogenesis of cervical cancer. Coinfection with different human viruses has been also reported in oropharyngeal and oral cavity cancers [100]. Although there are yet no functional data available concerning possible interplay and helper functions of HPyVs with other viruses in human cancers, it might be interesting to study this for HPyV6 and 7.

### Further studies to elucidate the role of HPyV6 and 7 in etiopathogenesis of human cancers

The HPyV6 and 7 contributions to human tumorigenesis are not yet elucidated. Therefore, several important questions regarding the pathogenesis of both viruses need to be addressed. For example, how to confirm the interaction of HPyV6 and HPyV7 in the inactivation of p53 and retinoblastoma protein (pRb), or in the transformation function. Rozenblatt-Rosen et al. namely proposed this mechanism in which HPyV6 and 7 both LTAg and sTAg proteins to bind p53 and inactivate it leading to tumorigenesis [32]. MCPyV is the only known HPyV that is found to be clonally integrated into MCC cases, in particular, the binding of truncated LTAg to the pRb was found to maintain the growth of MCC in MCPyV-positive cell lines [3, 83, 106]. Furthermore, SV40, which is a non-HPyV, was reported to bind p53 and Rb and inactivate their function [107–109]. In addition, one study has shown that HPyV6 sTAg is capable to inactivate the tumor suppressor protein phosphatase 2A (PP2A) that results in hyperphosphorylation of the MEK-ERK pathway and leads to abnormal proliferation [110, 111].

The presence of HPyV6- and 7-DNA and their association with human malignancies have commonly been tested by PCR and qPCR. However, the conclusions that can be drawn from these studies restricting their level of evidence to the proof of viral nucleic acids in homogenized tissue extracts are unfortunately very limited. The meaning of the presence of viral nucleic acids must remain inconclusive because these findings cannot be interpreted on the single-cell level in the histomorphological context of the specific disease. In addition, only limited reliable data are available on the viral protein expression, because the detection of antibodies by IHC in FFPE tissues are lacking. A possible screening approach might start with the use of the PAb416 antibody followed by specific HPyV6 and 7 PCR. In addition, studies definitely are needed in order to investigate next to the presence of HPyV6-DNA and 7-DNA, if these viruses are integrated into the tumor genome and possibly carry tumor-specific mutations within the viral helicase of LTAg in analogy to MCPyV [15, 17]. This will e.g. also help to understand the role of HPyV6 and 7 in skin carcinogenesis as a possible co-factor of UV-radiation as it is discussed for HPV5 and 8 association skin carcinogenesis [49, 112].

## Conclusion

In this review, we aimed to comprehensively analyze the literature for a possible role of the two closely related HPyV6 and 7 in human diseases and cancers. The seropositivity of HPyV6 was found to be higher than HPyV7 and both increased with age. The etiopathogenetical contribution of these viruses to human cancers remains unclear. Studies revealed that low HPyV6 and HPyV7 prevalence in non-neoplastic tumors resembled those in malignancies. Interestingly, HPyV6 prevalence was higher in skin malignancies than that of HPyV7. In contrast, HPyV7 was more frequently detected in non-cutaneous malignancies compared to HPyV6.

Based on sero- and tissue-prevalence HPyV6 and 7 remain important putative candidates for contributing to the etiopathogenesis of human disease, including skin cancers. There is an urgent need to further define their presence within cutaneous lesions e.g., keratoacanthoma, squamous cell carcinoma, and basal cell carcinoma, and to apply additional molecular techniques to unravel the molecular basis of virus-host interactions and assess a possible role of viral integration or mutation of the LTAg in skin cancer development. The same applies for HPyV7-associated tumors e.g., cholangiocellular carcinomas or thymomas. Further studies are needed to understand the contribution of HPyV6 and HPyV7 to human cancers.

## Data Availability

Not applicable.

## Abbreviations

HPyVs: Human polyomaviruses
HPyV6: Human polyomavirus 6
HPyV7: Human polyomavirus 7
PyV’s: Polyomaviruses
MCPyV: Merkel cell polyomavirus
MCC: Merkel cell carcinoma
VP1: Viral protein 1
VLP: Virus-like particles
ELISA: Enzyme-linked immunosorbent assays
GST: Glutathione S-transferase
HPV: Human papillomavirus
ALHE: Angiolymphoid hyperplasia with eosinophilia
FISH: Fluorescence in situ hybridization
FFPE: Formalin-fixed-paraffin-embedded
MM: Malignant melanoma
BCC: Basal cell carcinoma
SCC: Squamous cell carcinoma
IHC: Immunohistochemistry
CTCL: Cutaneous T-cell lymphomas
CCA: Cholangiocarcinoma
LTAg: Large tumor antigen
